# Metagenomic Sequencing for Diagnosing Listeria-Induced Rhombencephalitis in Patient and Contaminated Cheese Samples: A Case Report

**DOI:** 10.3390/ijms26020655

**Published:** 2025-01-14

**Authors:** Katarina Resman Rus, Martin Bosilj, Tina Triglav, Matjaž Jereb, Mateja Zalaznik, Maša Klešnik, Danilo Češljarac, Mojca Matičič, Tatjana Avšič-Županc, Tomaž Rus, Misa Korva

**Affiliations:** 1 Institute of Microbiology and Immunology, Faculty of Medicine, University of Ljubljana, 1000 Ljubljana, Slovenia; katarina.resman@mf.uni-lj.si (K.R.R.);; 2 Clinic for Infectious Diseases and Febrile Illnesses, University Medical Centre Ljubljana, 1000 Ljubljana, Slovenia; 3 Faculty of Medicine, University of Ljubljana, 1000 Ljubljana, Slovenia; 4 Department of Neurology, University Medical Centre Ljubljana, 1000 Ljubljana, Slovenia

**Keywords:** rhombencephalitis, *Listeria monocytogenes*, consumption of infected cheese, metagenomic next generation sequencing

## Abstract

Among the various causes of rhomboencephalitis, *Listeria monocytogenes* infection is the most common. However, conventional microbiological methods often yield negative results, making diagnosis challenging and leading to extensive, often inconclusive, diagnostics. Advanced molecular techniques like metagenomic next-generation sequencing (mNGS) offer a powerful and efficient approach to pathogen identification. We present a case of life-threatening rhomboencephalitis in a 32-year-old immunocompetent patient where extensive microbiological, immunological, and biochemical tests were inconclusive. Given the patient’s consumption of unpasteurized homemade cheese, neurolisteriosis was suspected, and mNGS was employed on clinical samples (CSF, serum, urine) and the food source to identify the pathogen. mNGS detected *L. monocytogenes* in both patient samples and the cheese. Mapping reads were distributed across the genome, with 18.9% coverage in clinical samples and 11.8% in the cheese sample. Additionally, the Listeriolysin (*hlyA*) gene was detected with 22.3% coverage in clinical samples and 12.3% in the food source, confirming neurolisteriosis. The patient fully recovered following antibiotic treatment. This case underscores the importance of mNGS in diagnosing CNS infections when conventional methods yield negative results, and supports its inclusion in diagnostic protocols for suspected neurolisteriosis, particularly when traditional methods prove inadequate.

## 1. Introduction

Rhomboencephalitis, an inflammation of the brainstem and cerebellum, manifests as encephalopathy, cranial nerve palsies, long-tract deficits and cerebellar dysfunction, leading to significant morbidity and mortality. Among the various infectious, autoimmune and paraneoplastic causes, *Listeria monocytogenes*, a Gram-positive, food-borne pathogen, is the most common etiology [1,2]. Timely and accurate diagnosis is critical since different, potentially life-threatening causes require specific treatments. Conventional microbiological methods often fail to detect *L. monocytogenes* in the cerebrospinal fluid (CSF) due to low bacterial counts or effective prior antibiotic therapy [3]. Metagenomic next-generation sequencing (mNGS) can improve pathogen identification in suspected central nervous system (CNS) infections, even when conventional diagnostic tests are negative, as previously described in patients with initially undiagnosed or misdiagnosed neurolisteriosis [4,5,6]. In this report, we describe the use of mNGS to identify *L. monocytogenes* in patient fluids and linked food source samples, revealing the cause of life-threatening rhombencephalitis that was overlooked by conventional microbiological diagnostics.

## 2. Case Presentation

A 32-year-old surgeon presented to the emergency neurology clinic with a severe headache that had persisted for two days, accompanied by diplopia, dysarthria, and dysphonia. Clinical examination revealed left-sided miosis and ptosis, gaze-evoked nystagmus, mild left-sided hemiparesis, appendicular and gait ataxia, and mild somnolence. The patient was afebrile, with no meningeal signs being present and no other abnormalities noted on physical examination, including the absence of skin or mucosal changes indicative of vasculitis or Behçet disease.

**Figure 1:**
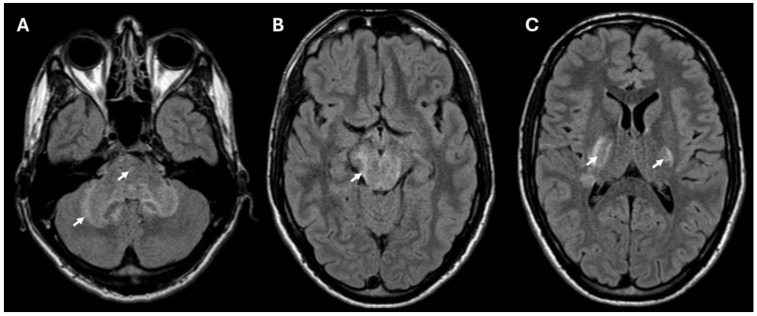
MRI (FLAIR sequence) showing hyperintense lesions consistent with rhombencephalitis in the cerebellum and pons (**A**), and lesions in the midbrain (**B**) and bilaterally in the posterior part of the internal capsule (**C**). The lesions in the brain are marked with arrows.

Brain MRI revealed T2/FLAIR hyperintense lesions in the posterior internal capsule, cerebral and cerebellar peduncles, midbrain, pons, and anterior medulla (Figure 1) suggestive of rhombencephalitis. There were no signs of restricted diffusion on DWI, and only minimal border contrast enhancement was observed. Cerebrospinal fluid (CSF) analysis showed elevated leukocytes (349 × 10^6^/L) with a predominance of polymorphonuclear cells (326 × 10^6^/L), high proteins (2.42 g/L) and a decreased glucose level. C-reactive protein (CRP) concentration in the blood was elevated (91 mg/L) as well as the leukocyte count (15 × 10^9^/L) with a neutrophyl predominance (80.7%).

During observation in the emergency room, the patient’s condition deteriorated, with worsening somnolence. Immediate empirical treatment with acyclovir, ampicillin, and cefotaxime was initiated, and the patient was admitted to the intensive care unit of the Clinic for Infectious Diseases, University Medical Centre Ljubljana.

Extensive microbiological diagnostic tests in CSF were negative, including bacterial culture, PCR for *L. monocytogenes*, herpes simplex virus 1 and 2, varicella-zoster virus, cytomegalovirus, human herpesvirus 6, enteroviruses, parechoviruses, Toscana virus and West Nile virus, as well as broad-range bacterial PCR. Further, the relevant diagnostic tests excluded Lyme borreliosis, tick-borne encephalitis virus, respiratory viruses, tuberculosis, hepatitis viruses, HIV, Whipple disease, neurosyphilis, *Toxoplasma gondii*, and other neurotropic parasites. Additionally, tests for antibodies against surface neuronal proteins and antiganglioside antibodies were negative.

In the following days, under the introduced antimicrobial treatment the patient’s clinical condition partially improved. However, the follow-up brain MRI performed two weeks after admission showed regression of T2/FLAIR hyperintense lesions in the brainstem and internal capsule, but a new hyperintense lesion was observed subcortically in the left frontoparietal region. The aforementioned microbiological tests were repeated and remained negative, including culture and *L. monocytogenes* real-time PCR assay (LightMix^®^ Modular *Listeria monocytogenes*, Roche). Additional investigations were conducted, with CSF cytology and flow cytometry excluding atypical cells. The CD4/CD3 T-cell ratio was only mildly and nonspecifically elevated. Tests for anti-aquaporin-4 and anti-MOG antibodies, as well as immunoserological rheumatic tests, were also negative. To exclude potential paraneoplastic disease, a whole-body FDG-PET scan was performed in addition to tumor marker tests in blood; both were unremarkable.

Despite several negative molecular and culture results for *L. monocytogenes*, neurolisteriosis was strongly suspected due to the clinical course and the patient’s history of consuming homemade unpasteurized cheese 20 days prior to disease onset, so empiric ampicillin treatment was continued.

As the pathogen could not be detected by conventional diagnostic methods, the mNGS approach was used for clinical samples (CSF, serum and urine) and samples of linked cheese.

## 3. Metagenomic Sequencing: Materials and Methods

Three clinical samples (CSF, serum, urine) and one sample of cheese consumed by the patient prior to symptom onset were included in the mNGS analysis. Nucleic acid was extracted from 400 μL of CSF and serum using EZ1 Virus Mini Kit v2.0 (Qiagen, Hilden, Germany) and from 2 mL of urine using QUICK-DNA/RNA Viral kit (Zymo Research, Irvine, CA, USA) following the manufacturer’s protocol.

For the cheese sample, 16 subsamples were prepared from different sections of the frozen cheese. DNA was isolated using the Reagent mericon DNA bacteria plus kit (Qiagen) after sample enrichment in 90 mL tryptone-soy broth, in which 10 g of cheese was incubated at 30 °C for 48 h. Additionally, DNA was isolated using the modified CTAB (cetyltrimethylammonium bromide) method, which was conducted from 200 mg cheese samples according to the previously published protocol [7].

For metagenomic Next-Generation Sequencing (mNGS), two libraries were prepared for each sample. The first library was prepared directly from isolated nucleic acids. For the second library, samples underwent pretreatment with the Turbo DNA-free kit (Thermo Fisher Scientific Inc., Waltham, MA, USA) and the Sequence-Independent, Single-Primer-Amplification (SISPA) method [8] to enrich RNA reads. NGS libraries were prepared using the Nextera XT library preparation kit (Illumina, San Diego, CA, USA) and were sequenced on NextSeq 550 using the NextSeq 500/550 High Output Kit v2.0 (300 cycles).

After sequencing, short paired-end (PE) raw reads were analyzed with MetaAll (https://github.com/NGS-bioinf/MetaAll, accessed on 24 November 2023) workflow [9]. Visualization of mapping results was performed using R statistical software (version 4.2.3, R Foundation for Statistical Computing, Vienna, Austria). In order to obtain a more reliable detection, the results of all clinical samples and the results of all cheese subsamples were combined.

## 4. Results

**Figure 2:**
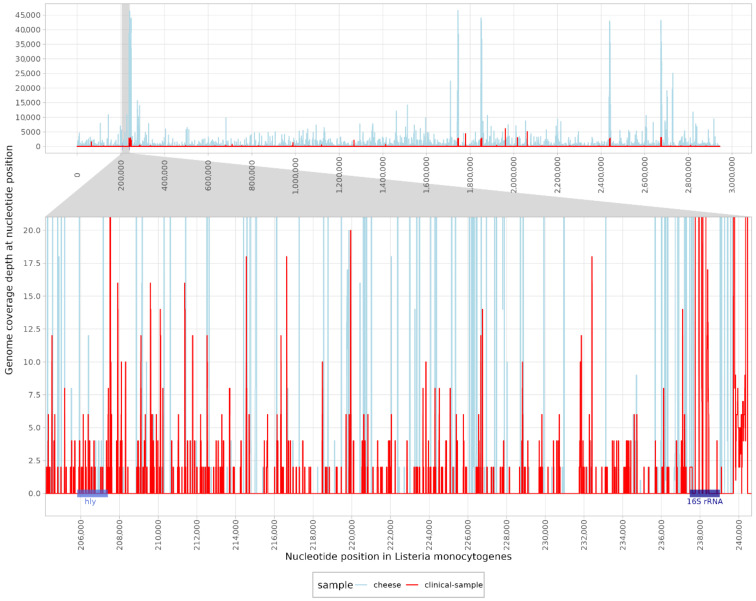
Mapping reads of a clinical (red) and a cheese sample (blue) to the reference genome of *Listeria monocytogenes* (NC_003210.1). The enlarged region shows the coverage of the *hly* (listeriolysin) gene and the 16S rRNA region.

A total of 67,811,301 raw paired-end (PE) reads were classified for clinical samples and 240,913,154 raw PE reads were sequenced for cheese samples. When classifying the reads obtained from the clinical sample, 12 reads were assigned to the genus Listeria. In contrast, 8056 reads and 6003 unique k-mers were classified as *L. monocytogenes*. The high number of unique k-mers indicated reliable detection with a good distribution of reads across the genome. Contig classification revealed nine contigs in the clinical samples and three contigs in the cheese sample, which were identified as *L. monocytogenes*. The mapping reads were distributed across the genome, with a coverage of 18.9% in the clinical and 11.8% in the cheese samples (Figure 2) using the reference genome (NC_003210.1). Moreover, the listeriolysin (*hlyA*) gene was detected with 22.33% coverage in clinical and 12.33% coverage in cheese samples. Other pathogens that have been described as possible causative agents of rhombencephalitis have not been identified, as both classification methods were negative (Table 1).

The mNGS results in combination with the clinical presentation of the disease, the epidemiologic data and the improvement of the patient after initiation of antibiotic therapy against *L. monocytogenes* highly suggest that the rhombencephalitis was caused by the consumption of homemade unpasteurized cheese infected with *L. monocytogenes*. The patient was treated with intravenous ampicillin for six weeks, and discharged with slight diplopia and isolated memory deficits, both of which resolved over the following months, leading to a complete recovery.

## 5. Discussion

In the presented case, mNGS was crucial for the identification of *L. monocytogenes* as the cause of life-threatening rhombencephalitis in an immunocompetent young patient and for the confirmation of its presence in the linked food source.

Despite negative culture and specific molecular testing, neurolisteriosis was clinically suspected due to symptoms onset following the consumption of unpasteurized homemade cheese [10]. The detection rate of *L. monocytogenes* in routine CSF and blood cultures is low, with positivity in only 41% and 61% of cases, respectively [11,12]. Therefore, antibiotic treatment is necessary in suspected cases, even with negative test results. In line with guidelines, empiric antibiotic therapy for *L. monocytogenes* was initiated immediately after diagnosing rhombencephalitis [13]. Immediate antibiotic therapy contributed significantly to the patient’s rapid and complete recovery, while delays in appropriate treatment can lead to severe sequelae in about 60% of survivors [14].

A mNGS procedure was performed to detect the genome of potential pathogens. Previous research highlights the advantages of mNGS over conventional gold standard methods for CNS infections, demonstrating a significantly higher positive rate compared to culture (100% vs. 16%) [3,15]. *L. monocytogenes* was detected in CSF, serum and urine using mNGS. It has been shown that mNGS is reliable even when the number of reads detected as *L. monocytogenes* is low [6,12,16]. Furthermore, the source of infection was confirmed by an even higher number of reads detected as *L. monocytogenes* in a cheese sample. Additionally, the pathogenic potential of the detected *L. monocytogenes* was confirmed by the detection of the *hly* gene in both the clinical and food samples, although a direct comparison of the sequences was not possible.

Despite its significant potential, mNGS may not yet be suitable for routine diagnostics due to its high costs, long processing times and lack of standardized protocols. Currently, its application is limited to select cases that require close collaboration between clinicians, microbiologists, and bioinformaticians [5]. However, technological advances promise to increase the utility of mNGS and position it as a valuable complement to classical microbiological diagnostics in the future. Nonetheless, mNGS can be a valuable tool for the diagnosis of neurolisteriosis, even after prior antibiotic treatment.

## 6. Conclusions

This case provides evidence that mNGS can represent an invaluable tool for the clinical diagnosis of rhombencephalitis. It can detect various genes in the *L. monocytogenes* genome and is less affected by prior antibiotic treatment. However, knowledge of the configurable bioinformatic parameters is crucial for the correct interpretation of the results. Although mNGS is an expensive technology, it can accurately identify potential pathogens, reducing delays in diagnosis and treatment. Early and accurate diagnosis with mNGS may prevent unnecessary testing and ultimately reduce overall diagnostic costs.

## Figures and Tables

**Table 1:** Mapping statistics of reads to selected reference genomes for clinical samples.

Sample	Pathogen (Reference)	Reads Number	Bases Coverage	Coverage (%)	Mean Depth
CSF	***Listeria monocytogenes*** (NC_003210.1)	**9376**	**56,108**	**1.90**	**0.0580**
	Epstein–Barr virus (NC_007605.1)	272	1966	1.14	0.0296
	enterovirus 71 (U22521.1)	12	110	1.48	0.0294
	human herpesvirus 6 (NC_000898.1)	511	2684	1.65	0.0540
	herpes simplex virus 1 (NC_001806.1)	137	1004	0.66	0.0170
Serum	***Listeria monocytogenes*** (NC_003210.1)	**13,617**	**88,060**	**2.99**	**0.0845**
	Epstein–Barr virus (NC_007605.1)	352	2352	1.37	0.0377
	enterovirus 71 (U22521.1)	6	57	0.77	0.0152
	human herpesvirus 6 (NC_000898.1)	584	3904	2.41	0.0634
	herpes simplex virus 1 (NC_001806.1)	124	907	0.59	0.0146
Urine	***Listeria monocytogenes*** (NC_003210.1)	**64,747**	**317,859**	**10.79**	**0.3971**
	Epstein–Barr virus (NC_007605.1)	1498	8538	4.97	0.1611
	enterovirus 71 (U22521.1)	44	333	4.49	0.1057
	human herpesvirus 6 (NC_000898.1)	2976	13,512	8.33	0.3122
	herpes simplex virus 1 (NC_001806.1)	546	3106	2.04	0.0643

CSF—cerebrospinal fluid.

## Data Availability

The data that support the findings of this study are available from the corresponding authors, T.R. and M.K. (Misa Korva), upon reasonable request.
